# A Homozygous Loss-of-Function Mutation in *MSH5* Abolishes MutSγ Axial Loading and Causes Meiotic Arrest in NOA-Affected Individuals

**DOI:** 10.3390/ijms23126522

**Published:** 2022-06-10

**Authors:** Chenjia Gong, Tanveer Abbas, Zubair Muhammad, Jianteng Zhou, Ranjha Khan, Hui Ma, Huan Zhang, Qinghua Shi, Baolu Shi

**Affiliations:** 1 The First Affiliated Hospital of University of Science and Technology of China, University of Science and Technology of China, Hefei 230001, China; gcj123@mail.ustc.edu.cn (C.G.) tanveerabbas@mail.ustc.edu.cn (T.A.); zubair512@mail.ustc.edu.cn (Z.M.); zjt13@mail.ustc.edu.cn (J.Z.); rhanjha@mail.ustc.edu.cn (R.K.); clsmh@ustc.edu.cn (H.M.); zhh1985@ustc.edu.cn (H.Z.); 2 The CAS Key Laboratory of Innate Immunity and Chronic Disease, University of Science and Technology of China, Hefei 230027, China; 3 School of Basic Medical Sciences, Division of Life Sciences and Medicine, University of Science and Technology of China, Hefei 230027, China; 4 Biomedical Sciences and Health Laboratory of Anhui Province, University of Science and Technology of China, Hefei 230027, China

**Keywords:** male infertility, non-obstructive azoospermia, meiotic arrest, recombination, synapsis, *MSH5*, MutSγ

## Abstract

Non-obstructive azoospermia (NOA), characterized by spermatogenesis failure and the absence of sperm in ejaculation, is the most severe form of male infertility. However, the etiology and pathology between meiosis-associated monogenic alterations and human NOA remain largely unknown. A homozygous *MSH5* mutation (c.1126del) was identified from two idiopathic NOA patients in the consanguineous family. This mutation led to the degradation of *MSH5* mRNA and abolished chromosome axial localization of MutSγ in spermatocytes from the affected males. Chromosomal spreading analysis of the patient’s meiotic prophase I revealed that the meiosis progression was arrested at a zygotene-like stage with extensive failure of homologous synapsis and DSB repair. Therefore, our study demonstrates that the *MSH5* c.1126del could cause meiotic recombination failure and lead to human infertility, improving the genetic diagnosis of NOA clinically. Furthermore, the study of human spermatocytes elucidates the meiosis defects caused by *MSH5* variant, and reveals a conserved and indispensable role of MutSγ in human synapsis and meiotic recombination, which have not previously been well-described.

## 1. Introduction

To date, about 48.5 million couples worldwide suffer from infertility, 50% of which are attributed to male factors [1]. It has been estimated that about 10% of infertile men are affected by azoospermia, which is the most severe form of male infertility [2]. Azoospermia is classified as obstructive azoospermia (OA) and non-obstructive azoospermia (NOA). By means of testicular biopsy and histological features, the etiology of NOA is classified into Sertoli-cell only, maturation arrest (including spermatocyte or spermatid stage arrest), and hypospermatogenesis [3]. With the widespread use of high-throughput approaches, whole genome sequencing (WGS) and whole exome sequencing (WES) become effective tools for exploring potentially pathogenic NOA-related single gene mutations and a number of genetic mutations were thus identified including *MEIOB*, *SPATA22*, *ZSWIM7*, and *C14ORF39* [4,5,6]. However, the causes of the majority of cases of the monogenic alterations in human NOA remain to be explored.

Meiosis specifically occurs in gametogenesis during which the genome halves via two consecutive rounds of cell division. During meiotic prophase, recombination is initiated by the SPO11-generated DNA double-strand breaks (DSBs) [7,8]. Subsequent end resection on DSB produces 3′ single-stranded DNA (ssDNA) overhangs [9]. The replication protein A (RPA) complex, consisting of RPA1, RPA2, and RPA3, coats the ssDNA to prevent ssDNA from degradation and the formation of secondary structures [10,11]. Loading of RPA on ssDNA is also a prerequisite for the recruitment of DNA recombinase DMC1 and RAD51 [12]. Later on, DMC1 and RAD51 gradually replace RPA and drive single strand invasion into homologous chromosomes to produce the nascent displacement loop (D-loop) [13,14,15,16,17]. As the D-loop extends, the second end capture takes place and results in the formation of double Holliday junctions (dHJs). Factors such as the MSH4/MSH5 complex and TEX11 are involved in stabilizing both nascent D-loop and dHJ structures [18,19,20]. As a consequence of meiotic recombination, the MLH1/MLH3 complex directs the resolution of dHJs to produce crossover(s) on every pair of homologs that serve as a bond to physically link homologous chromosomes and progressively turn into chiasmata, which facilitates accurate homolog bi-orientation on the spindle [21,22,23,24].

In eukaryotic cells, MSH4 and MSH5 belong to the MutS homolog (MSH) family, forming a heterodimer termed MutSγ, which plays specific roles at multiple steps in meiotic recombination [22,25,26]. In vitro and in vivo evidence revealed that MutSγ recognizes and binds to nascent D-loops and dHJs, stabilizes the recombinant intermediates, promotes crossover formation, and facilitates MLH1/MLH3 to convert these intermediates into mature crossovers [18,27]. *Msh5*^−/−^ mice exhibited chromosome synapsis failure and meiotic arrest at a zygotene-like stage, resulting in male and female sterility [28]. Variants in *MSH5* are associated with human primary ovarian insufficiency [29] and male infertility [30,31,32].

In the present study, we aimed to investigate the effect of the *MSH5* variant on human meiotic recombination, and further revealed the in vivo function of MutSγ in human meiosis. A homozygous *MSH5* mutation (c.1126del) was detected from a consanguineous Pakistan family by the WES combined with bio-informatic analyses. Sanger sequencing was conducted to validate that the *MSH5* mutation was carried by two idiopathic NOA-affected men in this family. We performed a testicular biopsy for one of the patients harboring the *MSH5* mutation, and determined the effect of the *MSH5* mutation in the patient’s testicular tissues by quantitative real-time PCR. To further elucidate the in vivo function of MutSγ in human meiosis, we conducted immunostaining using meiotic recombination markers on the patient’s spermatocyte spread slides.

## 2. Results

### 2.1. Clinical Characteristics of NOA-Affected Individuals from a Consanguineous Family

**Figure 1:**
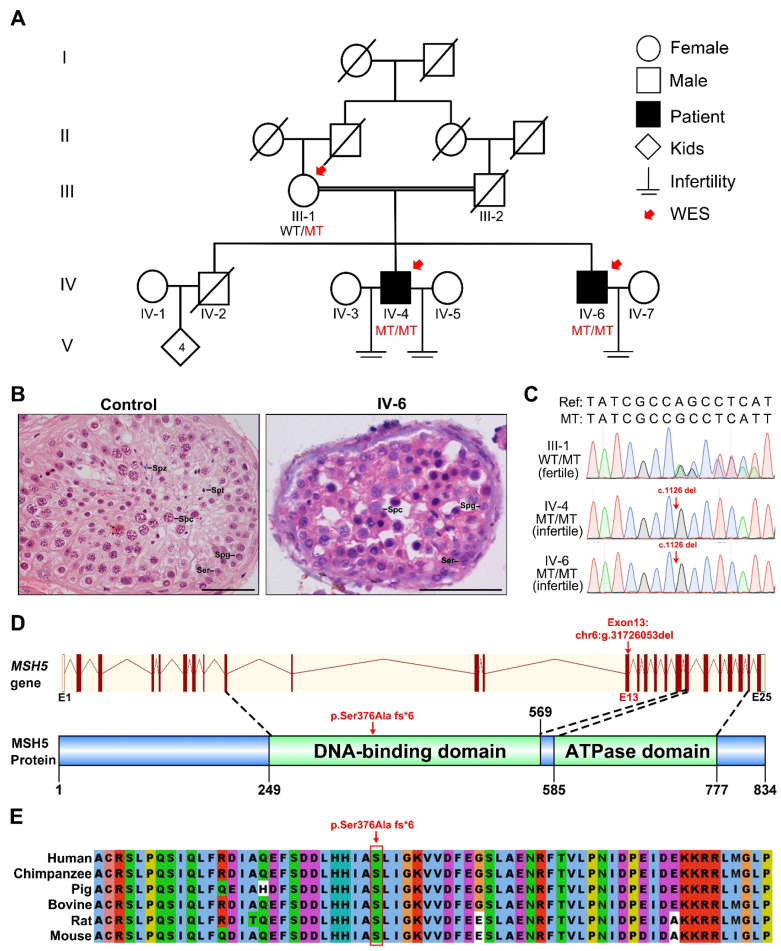
Identification of a homozygous *MSH5* mutation in the NOA individuals. (**A**) A consanguineous Pakistani family with two NOA individuals. WES was performed on members pointed by the red arrows. Double horizontal lines represent consanguineous marriages. Squares and circles denote male and female members, separately. Solid symbols indicate the infertile males, and open symbols denote unaffected members. Slashes represent deceased family members. (**B**) Testicular histological analyses of a male control with OA and the patient IV-6 by H&E staining. Spg, spermatogonia; Spc, spermatocyte; Spt, spermatid; Spz, spermatozoa; Ser, Sertoli cell. Scale bars indicate 50 μm. (**C**) Sanger sequencing chromatograms of the *MSH5* variant of this pedigree. The red arrows indicate the mutation site. (**D**) Schematic map of the mutation position in *MSH5* at the genomic and protein levels. The schematic gene structure was based on the Ensembl database (GRCh38, transcript ID: ENST00000375750.9). The red solid rectangles represent exons (exon 1 to 25), and the red lines represent introns. The schematic protein structure was based on the HPRD database (HPRD ID: 04544). (**E**) The conservation analysis of the *MSH5* mutant amino acid across different organisms.

We recruited a consanguineous Pakistan family with two idiopathic NOA-affected men (IV-4 and IV-6) who had no offspring after at least 10 years of marriage (Figure 1A and Table 1). Physical examinations indicated that they had normal stature, secondary sexual characteristics, and intellectual ability. Chromosome analysis revealed a normal karyotype, and no Y-chromosome microdeletions. Hormone measurements from IV-4 and IV-6 revealed that the levels of LH, prolactin, and testosterone were in the normal ranges, while the FSH level was higher than the reference value (Table 2). Testicular scrotal ultrasound examination reflected the normal testes size, shape, and outlines for IV-4 and IV-6 (Table 1). Semen analyses were conducted twice following the WHO guidelines and the results showed that both individuals had normal semen volume, but no sperm (Table 1). To further examine their reproductive status, testicular biopsies were performed on IV-6 and a male control with OA whose spermatogenesis was normal. Following H&E staining on the testicular sections, it was revealed that compared to those in the OA control, seminiferous tubules from IV-6 contained spermatogonia and spermatocytes, but were completely devoid of post-meiotic germ cells. These results indicate that the spermatogenesis was arrested at the spermatocyte phase in the patient (Figure 1B).

### 2.2. A Homozygous Frameshift Mutation in MSH5 Is Identified in Two Infertile Individuals

**Figure 2:**
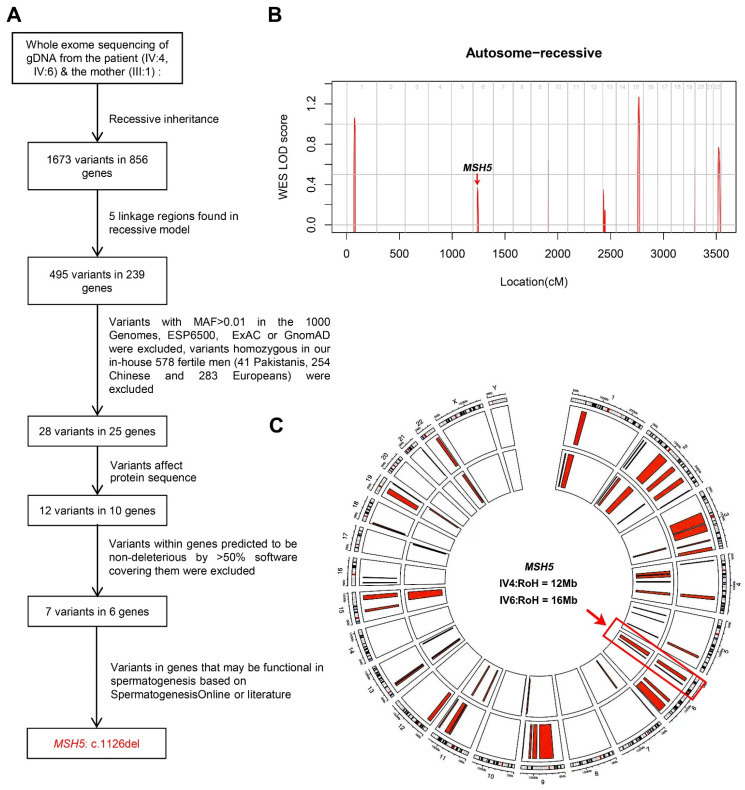
The workflow for the whole-exome sequencing data analysis. (**A**) The WES data analysis pipeline. MAF, minor allele frequency. (**B**) Genome-wide logarithm of the odd scores (LOD) using WES-derived genotypes for the family. (**C**) Homozygosity mapping analysis for individuals carrying the *MSH5* mutation.

To uncover the potential genetic cause of spermatocyte arrest in this family, WES was performed on all of the available family members including the two infertile individuals (IV-4 and IV-6) and their mother (III-1) (Figure 1A). The variants detected from the WES data were filtered with a series of criteria (Figure 2A). In brief, as the individuals were born to a consanguineous family, variants following recessive inheritance pattern in the sequenced family members were considered of priority. Linkage analysis was first performed and five linkage regions were identified (Figure 2B). The variants within these linkage regions and meeting the following conditions were given preference: (1) variants with minor allele frequency (MAF) <0.01 in the 1000 Genomes, ESP6500, ExAC, and Genome Aggregation Database; (2) variants affecting the protein sequence; (3) loss-of-function variants or potentially deleterious missense variants predicted by software including Sorting Intolerant From Tolerant, PolyPhen-2, and Mutation Taster; and (4) variants in genes expressed in the testis. Consequently, a 1-bp deletion in *MSH5* (c.1126del [p.Ser376Ala fs*6] [GenBank: NM_172166.4]) was screened out as a potentially pathogenic variant (Figure 2A). Homozygosity mapping analysis revealed that the *MSH5* variant was within long runs of homozygosity (RoH) regions in the two affected individuals (IV4: RoH = 12 Mb and IV6: RoH = 16 Mb) (Figure 2C). This *MSH5* variant was verified by Sanger sequencing and was confirmed to co-segregate with male infertility in this family (Figure 1C). The *MSH5* variant carried by IV-4 and IV-6 were further confirmed at the mRNA level in peripheral blood or testicular tissues, respectively (Figure S1). In addition, the 1-bp deletion in *MSH5* presumably causes a frameshift that changes the amino acid residue serine to alanine at position 376, which is located in the highly conserved DNA binding domain (Figure 1E and Figure S2), and introduces a premature translational termination of MSH5 at amino acid position 381 (Figure 1D). Therefore, we suggest that the identified homozygous *MSH5* c.1126del could be pathogenic for male infertility in this pedigree.

### 2.3. Dynamic Localization of MSH5 and MSH4 to Synaptonemal Complexes in Human

**Figure 3:**
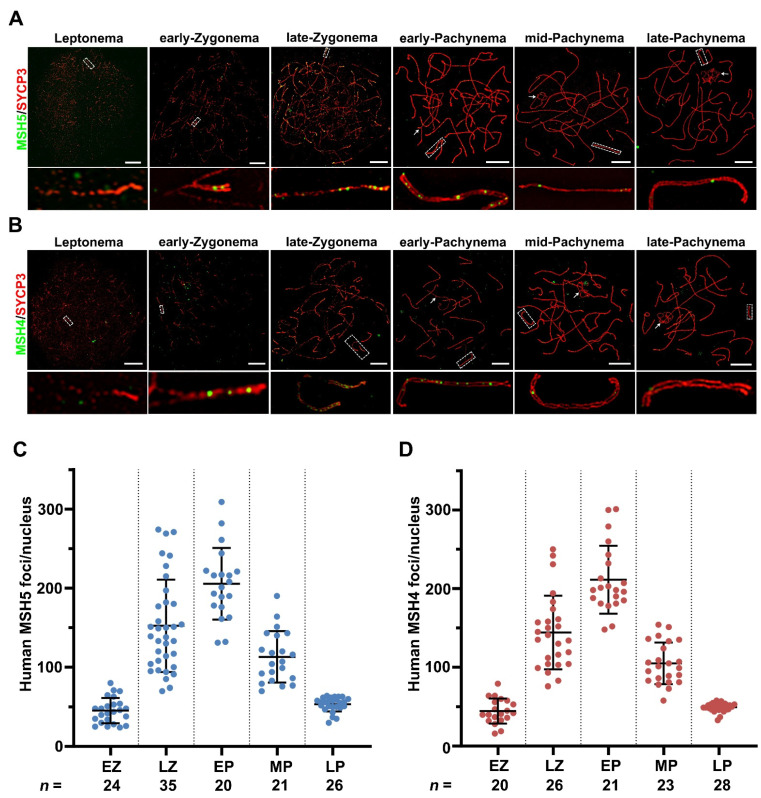
The super-resolution localization of human MSH5 and MSH4 on meiotic chromosomes. (**A**,**B**) The super-resolution images of human surface-spread spermatocytes showing the localization of human MSH5 (**A**) and MSH4 (**B**) on meiotic chromosomes. Early-, mid-, and late-pachynema were distinguished based on the XY morphology and SYCP3 staining pattern Arrowheads indicate the XY chromosomes. Enlarged view of the boxed individual chromosomes are shown below the overlay images. Scale bars, 10 μm. (**C**,**D**) Numbers of the MSH5 (**C**) and MSH4 (**D**) foci per nucleus at successive prophase stages (EZ, early-zygonema; LZ, late-zygonema; EP, early-pachynema; MP, mid-pachynema; LP, late-pachynema). Horizontal bars represent the mean ± SD. n represents the number of nuclei analyzed at EZ, LZ, EP, MP, and LP, respectively.

To acquire the dynamics of MutSγ in vivo, we analyzed the MSH5 and MSH4 localization patterns in spread spermatocytes from a patient diagnosed with OA by super-resolution structured illumination microscopy (SIM). In human spermatocytes, the MSH5 foci first appeared at leptonema, and began to associate with axes on synapsed regions at early-zygonema (45.33 ± 15.99 (SD); 24 nuclei; Figure 3A,C), supposing that MSH5 may function in the early steps of human meiotic recombination. Intensive MSH5 foci were observed at late-zygonema with an average of 152 foci per nucleus (152.3 ± 58.47 (SD); 35 nuclei; Figure 3A,C). The number of MSH5 foci peaked at early-pachynema (205.5 ± 45.34 (SD); 20 nuclei) and gradually declined from mid- to late-pachynema (mid-pachynema: 113.0 ± 32.47 (SD); 21 nuclei; and late-pachynema: 53.38 ± 9.073 (SD); 26 nuclei; Figure 3A,C). Similarly, the MSH4 foci presented throughout leptonema to late-pachynema with the same localization pattern as MSH5 (Figure 3B,D). Abundant MSH4 foci appeared at late-zygonema with an average of 144 foci per nucleus (144.1 ± 46.80 (SD); 26 nuclei) and peaked at early-pachynema (211.1 ± 43.15 (SD); 21 nuclei). By late-pachynema, only around 49 MSH4 foci were retained on the axes (49.36 ± 5.506 (SD); 28 nuclei). The distribution pattern of MSH5 and MSH4 in human spermatocytes suggested that they likely act together during meiosis prophase I in human males.

### 2.4. The Identified MSH5 Mutation Causes mRNA Degradation and Abolishes Axial Localization of MutSγ in the Patient

**Figure 4:**
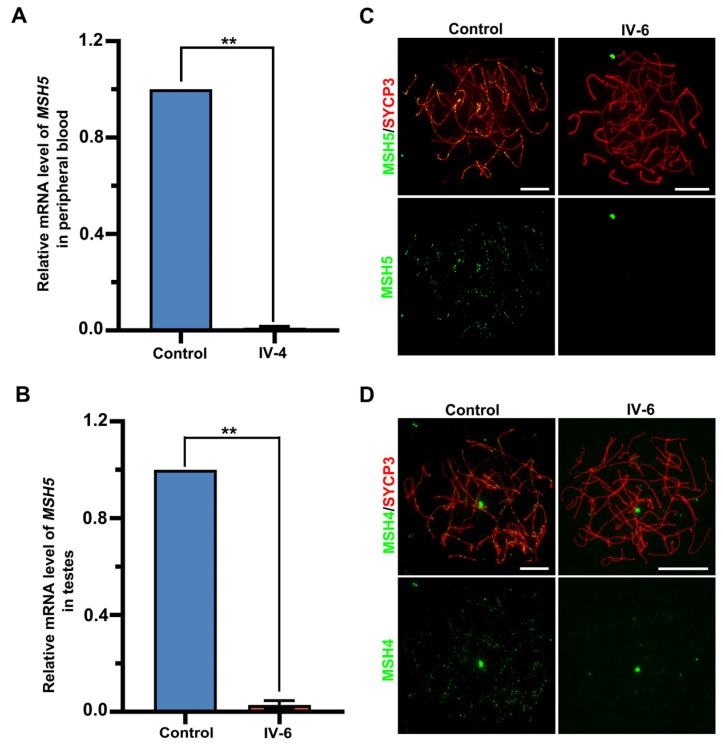
The effects of the MSH5 mutation on MSH5 expression and MSH4 loading. (**A**,**B**) Quantitative PCR analyses of the relative MSH5 expression in the blood samples (**A**) from the control and IV-4, and testicular samples (**B**) from the control and IV-6. ACTB served as an internal control. Data were obtained from two experiments and presented as mean ± SD. ** *p* < 0.01. Statistical significance is determined using the two-tailed t test. (**C**,**D**) Representative images of surface-spread spermatocytes from the control and IV-6 stained with antibodies against SYCP3 (red) and MSH5 (green) (**C**) or MSH4 (**D**). Scale bars, 10 μm.

To evaluate the effects of the identified mutation on *MSH5* expression, quantitative real-time PCR was performed and revealed that the level of *MSH5* mRNA in the peripheral blood of IV-4 was largely reduced to around 1.17% ± 0.57% (mean ± SD) compared to the control (Figure 4A). Similarly, only around 2.9% ± 1.8% (mean ± SD) of the *MSH5* mRNA expression was detected in the IV-6 testicular tissue compared to the control, indicating that *MSH5* mRNA was almost absent from the two affected individuals (Figure 4B). Further immunofluorescence staining on the spread spermatocytes with antibodies against SYCP3 and MSH5 confirmed a complete lack of MSH5 foci in all spermatocytes from IV-6 (Figure 4C).

MSH5 contains two regions (152–263 aa and 748–834 aa) that specifically interact with MSH4 to form the MutSγ heterodimer during meiosis [33,34,35]. To reveal the effect of the *MSH5* variant on MSH4 localization, we performed immunofluorescence staining using antibodies against SYCP3 and MSH4 on the human spread spermatocytes. Unlike the control spermatocytes in which the axes were decorated with abundant MSH4 foci, MSH4 signals were absent in all spermatocytes from IV-6 (Figure 4D). These results revealed that the homozygous *MSH5* c.1126del mutation is deleterious, provoking *MSH5* mRNA degradation and abolishing the axial binding of MutSγ in the patient’s spermatocytes.

### 2.5. Meiotic Arrest at Zygotene-like Stage with Synaptic Defects in the Patient

**Figure 5:**
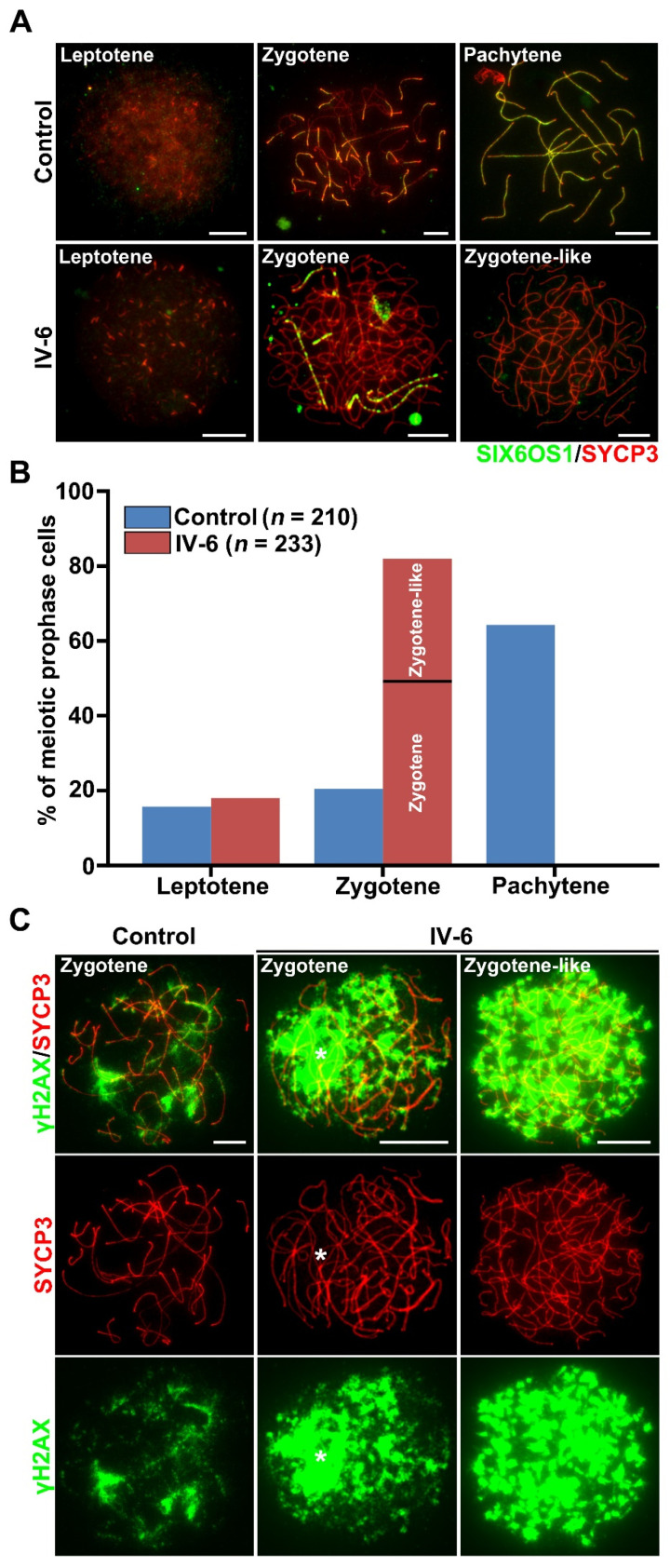
The failure of homolog synapsis and DSBs repair in the spermatocytes of patient. (**A**) Representative images of the surface-spread spermatocytes from the control and patient IV-6 stained with antibodies against SYCP3 (red) and SIX6OS1 (green). Scale bars, 10 μm. (**B**) Composition of the spermatocytes from control and IV-6. n, the number of spermatocytes analyzed. (**C**) Immunofluorescence staining of surface-spread spermatocytes from the control and IV-6 with antibodies against SYCP3 (red) and γH2AX (green). Asterisk indicates intensive γH2AX signal on the unsynapsed region. Scale bars, 10 μm.

To analyze the meiotic progression in more detail, immunofluorescent staining of SYCP3 and SIX6OS1, a central element of the synaptonemal complex, was conducted. In the control spermatocytes, three sub-stages of meiotic prophase I, leptotene, zygotene, and pachytene were identified, and the pachytene spermatocytes constituted approximately 63% of the total populations. Strikingly, no pachytene cells were detected in the patient’s samples, instead, over 80% of cells were zygotene (and-like) cells, which were significantly higher than the frequency of zygotene cells in the control samples (Figure 5B). Based on the extent of synapsis, two deviant types of zygotene (and-like) cells in the patient could be distinguished: (I) Type I cells with linear SIX6OS1 signals detected along paired chromosome axes, indicating partial synapsis; and (II) type II cells had linear lateral elements but were completely devoid of the central element, indicating the absence of synapsis; these cells were termed as zygotene-like and accounted for 32.62% of the total spermatocytes in the patient (Figure 5A,B). These results collectively indicate that the patient harboring *MSH5* c.1126del had meiosis arrest at a zygotene-like stage, accompanied with severe synapsis failure between homologous chromosomes.

### 2.6. Failure of Meiotic DSB Repair in Spermatocytes from the Patient

MutSγ is known to bind and stabilize the recombinant intermediates to promote recombination-based DSB repair [18,27,36]. To investigate meiotic DSB repair after the disruption of MutSγ, we performed immunofluorescence staining on the patient’s spread spermatocytes for SYCP3 and γH2AX, a marker of DNA breaks. Cloud-like γH2AX signals were distributed on some chromosome pairs in zygotene spermatocytes from the control. While in the patient, the overall intensity of γH2AX signals in the zygotene (and-like) cells were obviously higher than in the control zygotene cells. In the type I zygotene cells, strong γH2AX signals covered most of the unsynapsed homologs. In zygotene-like spermatocytes of the patient, the γH2AX signals were dispersed on every chromosome and appeared to be stronger than those in the type I zygotene cells (Figure 5C). These results demonstrate that the DSBs failed to be repaired in the patient’s spermatocytes.

## 3. Discussion

In this study, we identified a homozygous *MSH5* c.1126del in two NOA individuals from a consanguineous family, which triggered degradation of *MSH5* mRNA and loss of MSH5-MSH4 localization onto chromosome axes in the patients’ spermatocytes. A series of cytological studies in human spermatocytes revealed that the failure of MSH5-MSH4 localization on the chromosome axes impeded synapsis and meiotic DSB repair, resulting in meiotic arrest and azoospermia. Thus, our findings revealed that a novel biallelic variant of *MSH5* (c.1126del) is associated with human NOA.

MutSγ is well-known for its role in meiotic recombination in multiple organisms [28,37,38,39,40,41]. Previous studies on mouse models illustrated that loss of either *Msh5* or *Msh4* resulted in meiotic arrest at a zygotene-like stage, with aberrant pairing, insufficient synapsis, and a failure to complete DSBs repair [28,38,42]. Up to now, several variants in *MSH4* and *MSH5* have been reported in infertile men and women. However, the cytological consequence of MutSγ disruption in humans remains uncharacterized. Here, we showed that the loss-of-function mutation in *MSH5* caused meiotic defects similar to that in mice including the zygotene-like arrest and defects in synapsis and DSB repair. However, the extent of synapsis defects is different between the mouse and human mutants. In our patient, 32.62% spermatocytes formed the full (or almost full) length of the lateral axes but completely lacked central element signals, which was not described in the *Msh5* knockout mice [28]. It thus seems that MSH5 may serve a more important role in homolog synapsis in humans than in mice.

In mice, the degree of chromosome pairing and synapsis in *Msh4*^−/−^/*Msh5*^−/−^ mice is more similar to that in *Msh5*^−/−^ mice, and is less advanced than in *Msh4*^−/−^ mice [38]. Moreover, MSH4 foci on chromosome cores were dramatically decreased and became fainter in mutants with the disruption of the MSH5 ATP binding domain [43], suggesting that the MSH5 acts epistatic to MSH4 during mouse meiosis. Notably, in our patient, MSH5 expression was almost completely abolished, and we did not detect any MSH4 foci on the chromosome axes in all spermatocytes either, indicating that MSH4 recruitment or the association of MSH4 to the axes is dependent on MSH5. Referring to the mouse studies, we can infer that MSH5 is also likely to be a prerequisite for MSH4 recruitment and retention on the axes in human spermatocytes, and that the infertility outcome in the patient carrying the MSH5 mutation (c.1126del) is supposed to be caused by the abolished axial localization of both MSH4 and MSH5.

Recently, several pathogenic variants in *MSH5* have been found in NOA individuals [30,32]. Our study provides further supporting evidence on the pathogenicity of *MSH5* mutation in male infertility, improving the future genetic diagnosis of infertile individuals in clinic. More importantly, based on studies of human spermatocytes, we elucidate the meiosis defects caused by the *MSH5* variant and demonstrate a conserved and indispensable role of MutSγ in human synapsis and meiotic recombination, which have not been well-described previously.

## 4. Materials and Methods

### 4.1. Clinical Samples

A consanguineous Pakistani family was enrolled in this study. Written informed consent was received from all participants prior to the onset of the study. Semen analyses were performed according to the WHO guidelines.

### 4.2. Hormone Measurement

Before the blood was collected for hormone analysis, patients were advised to stop certain medications, which included not taking drugs such as oral steroids or estrogen supplements, TSH and testosterone level elevators, etc. The patient’s blood was collected at fasting condition (overnight) to avoid possible food interventions that can raise certain hormone levels in the blood. For hormone analysis, peripheral blood samples were collected from the available individuals using 5cc disposable sterile syringes (Becton Dickinson supplies Pakistan) and adopting standard protocols (WHO 2010). A total of 3 mL of peripheral blood was added to the serum separation tubes (SSTs) in the Tube with Gel and Clot Activator (VITREX Medical, Herlev, Denmark) for serum separation. Blood was kept in the SSTs and set for 30 min to 1 h at room temperature to clot before spinning and separating. This was then centrifuged for 5 min at approximately 1000× *g* and a clean pipette was used to remove the separated serum and immediately loaded to the COBAS e 411(Roche, Basel, Switzerland) special chemistry analyzer for hormone analysis.

### 4.3. Testicular Biopsy

#### 4.3.1. Pre-Operation Preparation of Patient

The patient was evaluated clinically for the procedure and anesthesia. The necessary hormone analyses, complete blood count (CBC), viral marker tests (HIV, HCV, HBS), and ultrasonographic testicular scans were performed. To control the hospital acquired infection, the required intravenous fluids and antibiotics were administered, the patient was kept nothing by mouth (NBM), and no foods and fluids were taken to keep the stomach empty.

#### 4.3.2. General Anesthesia (GA)

Before GA, the patient remained NBM for at least 10 h and was thoroughly evaluated clinically including weight, heart ECG, BP, and age factors.

#### 4.3.3. Surgical Removal of Testicular Tissue

The skin over the testicle was cleaned with a germ-killing (antiseptic) medicine. The area around it was covered with a sterile towel. A small surgical scrotal incision of 2–3 cm was made for the exposure of the tunica albuginea of the testis. A small incision in the capsule of the testicular of about 0.5 cm was made to obtain a biopsy of about 3 × 3 × 3 mm. For the adequate classification of spermatogenesis, while removing the tissue, it was ensured that it should contain at least 100 seminiferous tubules. At the same time, the tissue was not squeezed with forceps to not to disrupt the testicular tissue architecture and hamper the proper evaluation of the seminiferous tubules. The opening in the testicle was closed with a stich.

### 4.4. Whole-Exome Sequencing and Variant Filtration

Total genomic DNA was isolated from the peripheral blood of the study samples. Whole-exome capture and sequencing were performed using the AlExome Enrichment Kit V1 (iGeneTech, Beijing, China) and Hiseq2000 platform (Illumina, San Diego, CA, USA) following the standard procedures. The reads were aligned to the human genome reference assembly (hg19) using a Burrows–Wheeler Aligner with default parameters. Then, the Picard software (https://sourceforge.net/projects/picard/, download date: 24 September 2015) was employed to remove polymerase chain reaction duplicates. DNA sequence variants were called using the Genome Analysis Toolkit HaplotypeCaller (http://www.broadinstitute.org/gatk/, download date: 30 June 2018). Variants were filtered as previously described [44,45].

### 4.5. RNA Extraction, Quantitative Polymerase Chain Reaction (qPCR) and Reverse Transcription-Polymerase Chain Reaction (RT-PCR)

Human testicular tissue or peripheral blood were ground and solubilized in 1 mL TRIzol (Accurate Biology AG21101, Hunan, China). The total RNA was extracted according to the manufacturer’s protocol, as previously described [44], with minor modifications made as follows. A total of 200 µL chloroform was added to 1 mL TRIzol and centrifuged at 12,000× *g* for 15 min at 4 °C. After centrifugation, the upper clear aqueous phase was transferred to a 1.5 mL centrifuge tube, then 1 mL isopropanol was added and centrifuged at 12,000× *g* for 10 min at 4 °C. Discarding the supernatant, 1 mL of 75% ethanol was then added and centrifuged at 12,000× *g* for 10 min, and the obtained total RNAs were dissolved by RNase-free water. After denatured by heating at 56 °C in a metal bath for 5 min, the cDNAs were synthesized with the Prime Script RT Reagent Kit (TaKaRa RR047A, Shiga, Japan) according to the manufacturer’s protocols. The concentration and purity of cDNA were measured by NanoDrop 1000 Spectrophotometer (Thermo Fisher Scientific corporation, Waltham, MA, USA). The qPCR was performed with TransStart Top Green qPCR Super Mix (TransGen Biotech AQ132, Beijing, China) and a Step One Real-Time PCR System (Applied Biosystems, Thermo Fisher Scientific corporation, Waltham, MA, USA). We calculated the relative mRNA levels by normalizing to *ACTB* (MIM: 102630). The relative mRNA levels in the control were regarded as 1.0, and the fold change in the affected individual was compared to the control. Primer sequences are listed in Supplementary Table S1.

RT-PCR was conducted using templates from the peripheral blood or testicular cDNA, respectively. For the cDNA obtained from the peripheral blood of the male control and individual IV-4, PCR reactions were performed as follows: first round of PCR, 5 min at 94 °C, followed by 38 cycles of 15 s at 98 °C, 30 s at 58 °C, and 30 s at 72 °C; second round of PCR, 5 min at 94 °C, followed by 36 cycles of 15 s at 98 °C, 30 s at 55 °C, and 30 s at 72 °C. For the cDNA obtained from the testicular tissues of the male control and individual IV-6, PCR reactions were performed as follows: 3 min at 95 °C, followed by 38 cycles of 15 s at 95 °C, 15 s at 58 °C, and 30 s at 72 °C. The RT-PCR products were electrophoresed on a 2% agarose gel, followed by Sanger sequencing. Primer sequences are listed in Supplementary Table S1.

### 4.6. Hematoxylin and Eosin Staining

Human testicular tissues were fixed in Bouin’s solution overnight, then embedded into the paraffin, and sectioned at a 5 μm thickness. The H&E staining was conducted according to previously described protocols [6]. Briefly, the tissue slides were deparaffinized in xylene for 20 min, then rehydrated with gradient ethanol: 100%, 90%, 80%, and 50% (5 min for each concentration) and sequentially stained with hematoxylin for 5 min and eosin for 10 s. After stepwise dehydration with 50%, 80%, 90%, and 100% ethanol (2 min for each concentration) and transparency in xylene for 5 min, the tissue sections were sealed with neutral resin. The images were captured via a Nikon ECLIPSE 80i microscope (Nikon Instruments, Tokyo, Japan) with a DR-Ri1 camera and processed with NIS-elements Basic Research software (Nikon Instruments, Tokyo, Japan).

### 4.7. Spermatocyte Nuclear Surface Spreading and Immunofluorescence Staining

The surface-spreads of the human spermatocytes were prepared according to the previously described protocols [46]. Immunofluorescence staining was conducted according to the previously described protocols [6,47]. Briefly, slides were blocked with 3% non-fat milk in phosphate-buffered saline (PBS) for 10 min and then incubated with primary antibodies (mixed with 3% non-fat milk) overnight at 37 °C in a humidified chamber. After three washes in PBS containing 0.35% Triton X-100 (PBST), secondary antibodies (mixed with 3% non-fat milk) were applied for 1 h at 37 °C. Subsequently, the slides were washed three times in PBST and mounted with the Vectashield Medium (Vector Laboratories H1000, Newark, CA, USA). Images were captured with an Olympus BX61 microscope (Tokyo, Japan) with a CCD camera (QICAM Fast 1394, QImaging, Burnaby, BC, Canada) and processed with Image-Pro Plus software (Media Cybernetic, Rockville, MD, USA). Super-resolution imaging microscopy analysis was performed using a Nikon NSIM super-resolution microscope system and NIS-Elements 2 image processing software (Nikon Instruments, Tokyo, Japan). The antibodies are listed in Supplementary Table S2.

### 4.8. Statistical Analysis

GraphPad Prism Software (GraphPad Software, San Diego, CA, USA) was used to perform the statistical analysis. Statistical results were presented as mean ± SD in Figure 3C,D and Figure 4A,B. The Student’s *t*-test was used for all statistical analyses. The difference was considered as significant when the *p* values were <0.05.

## Figures and Tables

**Table 1:** The clinical characteristics of male individuals.

Subjects	IV-4		IV-6	
Basic Information				
Reproductive status	Infertility		Infertility	
Age (y) ^a^	37		44	
BMI	30.1		27.7	
Age (y) of marriage	18/26		23	
Semen Parameters ^b^	Sample 1	Sample 2	Sample 1	Sample 2
Semen volume (mL) ^c^	3.0	2.4	2.0	1.0
Sperm count (10^6^/mL) ^d^	0	0	0	0
Physical Examination				
External genitalia	Normal		Normal	
Secondary traits	Normal		Normal	
Ultrasonography				
Left testis size (cm)	3.9 × 2 × 1.7		3.52 × 2.78	
Right testis size (cm)	3.8 × 1.9 × 1.6		3.74 × 2.83	

^a^ Age at the manuscript submission. ^b^ Semen analyses were performed twice. ^c^ and ^d^ Reference values of semen volume and sperm count were >1.5 mL and >1.5 × 10^6^/mL, respectively, according to the WHO (6th Edition).

**Table 2:** The hormone concentrations.

Subjects	IV-4	IV-6	Reference Values ^a^
FSH (U/L)	15.33	33.93	0.95–11.95
LH (U/L)	4.12	10.31	0.57–12.07
Testosterone (ng/dL)	278.98	340.74	260–1000
Prolactin (ng/mL)	6.83	8.12	3.5–19.4

^a^ Reference values were suggested by the local clinical laboratory.

## Data Availability

The datasets used and/or analyzed during the current study are available from the corresponding author on reasonable request.

## Supplementary Materials

The following supporting information can be downloaded at: https://www.mdpi.com/article/10.3390/ijms23126522/s1.
