# Supplementary material for: A Homozygous Loss-of-Function Mutation in MSH5 Abolishes MutSγ Axial Loading and Causes Meiotic Arrest in NOA-Affected Individuals
Source: Int J Mol Sci. 2022 Jun 10;23(12):6522. doi: 10.3390/ijms23126522 (PMC9224491; doi:10.3390/ijms23126522)
Supplement: Supplementary file 1 [file ijms-23-06522-s001.zip › ijms-1739995-supplementary.pdf]

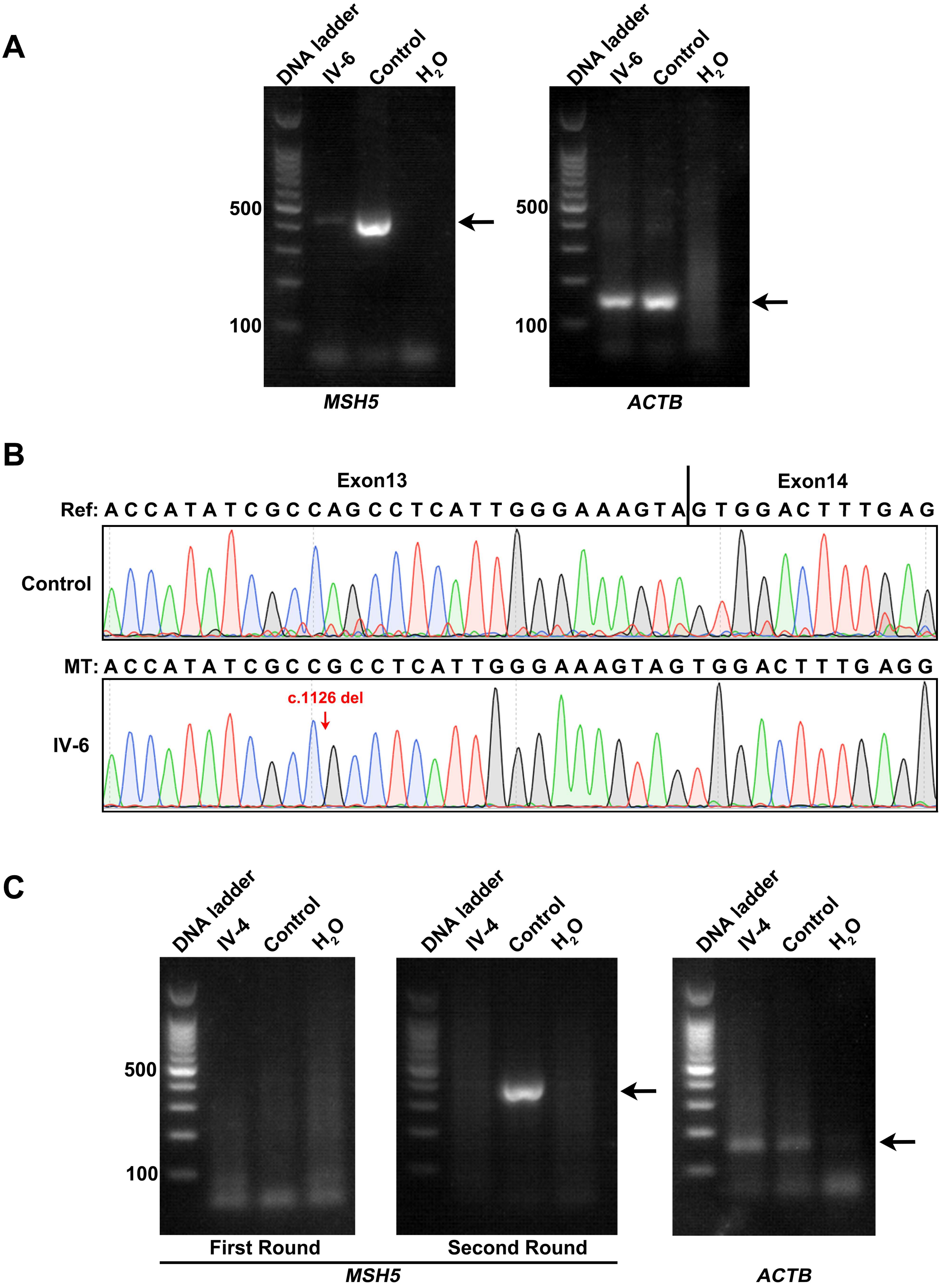

Figure S1. Detection of *MSH5* mutation at the cDNA level by RT-PCR and Sanger sequencing.
(A) RT-PCR analysis of testicular samples from a male control and the patient IV-6. *ACTB* served as a loading control. Arrows indicate targeted bands. (B) Sanger sequencing chromatograms of testicular cDNA products amplified from a male control and the patient IV-6. The red arrow indicates the 1-bp deletion in *MSH5*. (C) RT-PCR analysis of blood samples from a male control and the patient IV-4. *ACTB* served as a loading control. Arrows indicate targeted bands.

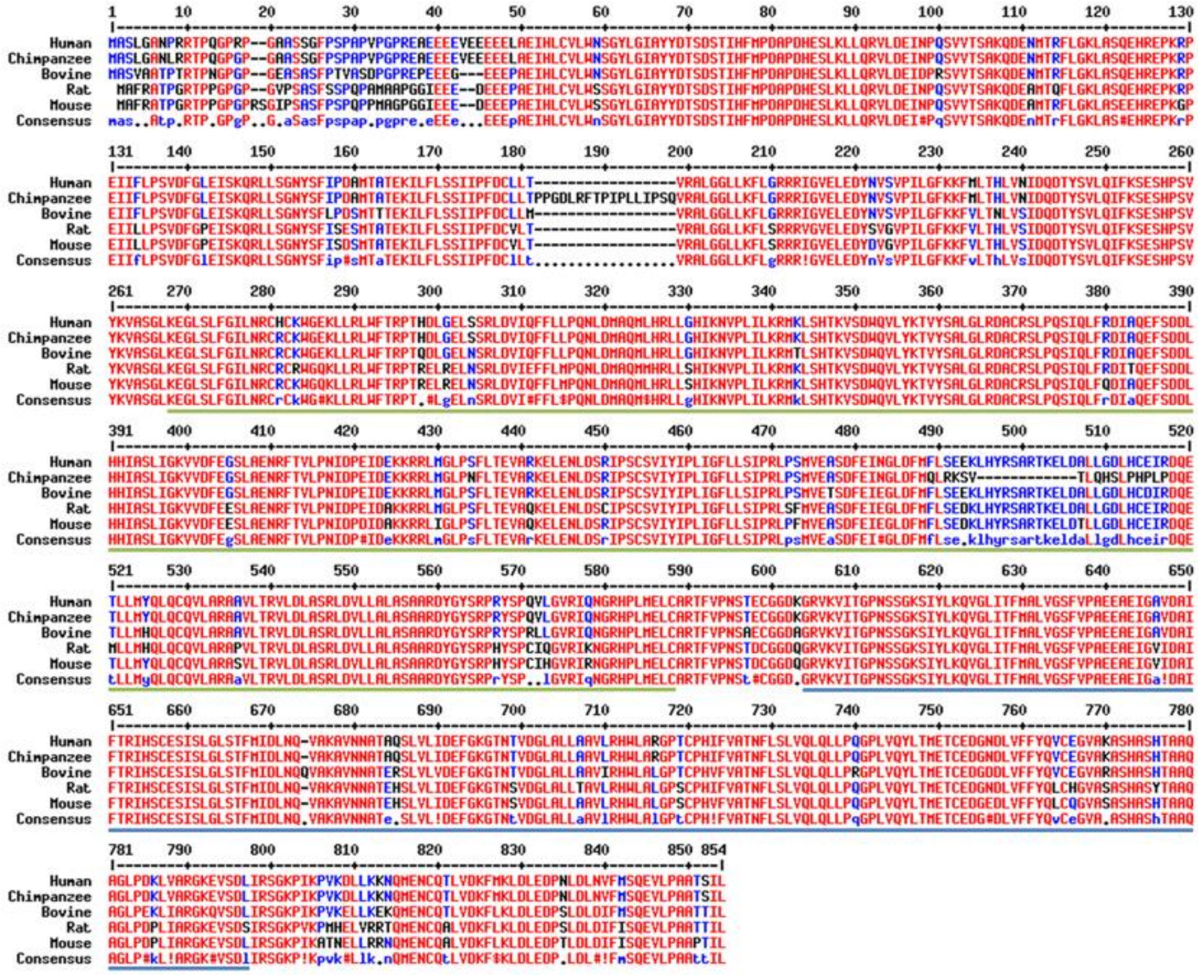

Figure S2. Alignment of MSH5 protein sequences across different organisms.

MSH5 residues that are identical among different species appear in red and as uppercase letters in the consensus line. Residues highly similar among different species are indicated by red symbols (!, any one of I and V; #, any one of N, D, and E). Unconserved residues are written in blue or as asterisks in the consensus line. Green line highlights the DNA-binding domain. Blue line highlights the ATPase domain. The alignment was performed using the online software MultAlin (http://multalin.toulouse.inra.fr/multalin/multalin.html).

Table S1. Primers used in this study.

Information	Name	Sequence(5'- 3')	Product size (bp)
Primers for human gDNA amplification and Sanger sequencing	hMSH5-720-F1	CCTATCACCACCTCAACCCG	Control: 403
	hMSH5-720-R1	TCCTGTTCCTCAGCTCTCCT	Mutant: 402
Primers for human cDNA amplification and Sanger sequencing	MSH5-720-cDNA-CF1	ATCTGGACATGGCTCAGATG	Control: 408
	MSH5-720-cDNA-CR1	ATGAAGGAATACGGGAGTCC	Mutant: 407
Primers used for RT-PCR (*MSH5*)	hMSH5-qRT-CF1	TACGAGTGCCAAACAGGATG	146
	hMSH5-qRT-CR1	CAGAAAGGAGGCGTTGTTTG	
Primers used for RT-PCR (*ACTB*)	hACTB-qPCR-F	AATGAGCTGCGTGTGGCTC	148
	hACTB-qPCR-R	ATAGCACAGCCTGGATAGCAAC	

Table S2. Antibodies used in this study.

Primary antibodies				
**Target**	**Host**	**Company**	**Catalog number**	**Dilution**
SYCP3	Mouse	Proteintech	66409-1-lg	IF: 1:100
MSH5	Rabbit	Sigma-Aldrich	HPA062688	IF: 1:50
MSH4	Rabbit	Abcam	ab58666	IF: 1:50
SIX6OS1	Guinea pig	Custom-made	-	IF: 1:100
γH2AX	Rabbit	NOVUS	NB100-384	IF:1:5000
**Secondary antibodies**				
**Target**	**Host**	**Company**	**Catalog number**	**Dilution**
Mouse (Alexa-488)	Goat	Molecular Probes	A-21121	IF: 1:100
Rabbit (Alexa-555)	Donkey	Molecular Probes	A31572	IF: 1:200
Guinea Pig (Alexa-555)	Goat	Thermofisher	A21435	IF: 1:200
